# Small interfering RNA mediated Poly (ADP-ribose) Polymerase-1 inhibition upregulates the heat shock response in a murine fibroblast cell line

**DOI:** 10.1186/1476-9255-8-3

**Published:** 2011-02-23

**Authors:** Rajesh K Aneja, Hanna Sjodin, Julia V Gefter, Basilia Zingarelli, Russell L Delude

**Affiliations:** 1 Departments of Critical Care Medicine and Pediatrics, University of Pittsburgh School of Medicine and Children's Hospital of Pittsburgh, Pittsburgh, PA 15213, USA; 2 Department of Critical Care Medicine, University of Pittsburgh School of Medicine Pittsburgh, PA 15213, USA; 3 Departments of Critical Care Medicine and Pathology, University of Pittsburgh School of Medicine, Pittsburgh, PA 15213, USA; 4 Division of Critical Care Medicine, Cincinnati Children's Hospital Medical Center and The University of Cincinnati College of Medicine, Cincinnati, Ohio 45229, USA

## Abstract

Poly (ADP-ribose) polymerase-1 (PARP-1) is a highly conserved multifunctional enzyme, and its catalytic activity is stimulated by DNA breaks. The activation of PARP-1 and subsequent depletion of nicotinamide adenine dinucleotide (NAD^+^) and adenosine triphosphate (ATP) contributes to significant cytotoxicity in inflammation of various etiologies. On the contrary, induction of heat shock response and production of heat shock protein 70 (HSP-70) is a cytoprotective defense mechanism in inflammation. Recent data suggests that PARP-1 modulates the expression of a number of cellular proteins at the transcriptional level. In this study, small interfering RNA (siRNA) mediated PARP-1 knockdown in murine wild-type fibroblasts augmented heat shock response as compared to untreated cells (as evaluated by quantitative analysis of HSP-70 mRNA and HSP-70 protein expression). These events were associated with increased DNA binding of the heat shock factor-1 (HSF-1), the major transcription factor of the heat shock response. Co-immunoprecipitation experiments in nuclear extracts of the wild type cells demonstrated that PARP-1directly interacted with HSF-1. These data demonstrate that, in wild type fibroblasts, PARP-1 plays a pivotal role in modulating the heat shock response both through direct interaction with HSF-1 and poly (ADP-ribosylation).

## Introduction

Poly (ADP-ribose) polymerase-1 (PARP-1) is a highly conserved chromatin bound enzyme [1,2] and plays an important role in DNA repair, gene transcription, cell-cycle progression, cell death, and maintenance of genomic integrity [3-5]. PARP-1 is activated by DNA breaks and cleaves nicotinamide adenine dinucleotide (NAD^+^) into nicotinamide resulting in ADP-ribose moieties; these moieties covalently attach to various acceptor proteins including PARP itself. The continued activation of PARP leads to depletion of its substrate NAD^+ ^with consequent depletion of ATP, energy failure and cell death [6].

In addition to its influence on chromatin structure and stability, recent studies indicate PARP-1 plays a role in gene-specific transcription [7-9]. PARP-1 regulates transcription by modifying chromatin-associated proteins and acts as a cofactor for transcription factors, most notably NF-κB and AP-1 [10,11]. Genetic deletion of PARP-1 attenuates tissue injury after ischemia and reperfusion, streptozocin-induced diabetes, endotoxic and hemorrhagic shock, heat stroke and localized colonic inflammation [12-19]. The benefits conferred by pharmacological inhibitors of poly (ADP-ribosylation) in diverse experimental disease models further reiterate the importance of PARP-1 as an important pharmacological target [20,21]

Oxidative injury and ATP depletion also leads to activation of heat shock factor (HSF)-1, a major transcription factor responsible for increased transcription of genes encoding heat shock proteins, particularly heat shock protein-70 [22,23]. HSP-70 provides cytoprotection from a variety of inflammatory insults, including oxidative stress, viral infections and ischemia-reperfusion injury [24,25]. Previously in an *in vivo *model of myocardial ischemia/reperfusion injury, we showed that cardioprotection conferred on PARP-1^-/- ^mice is associated with enhanced HSF-1 activity and increased expression of HSP-70 as compared to wild-type mice [26].

Similarly, Fossati *et al*. documented increased HSP-70 expression in murine PARP-1 deficient fibroblasts as compared to wild type fibroblasts [27]. In gene knockout cell lines, unexpected compensatory or redundant mechanisms develop in response to the missing gene and can confound experimental observations. To verify that the upregulation of the heat shock response in PARP-1 deficient mice is not a compensatory response to the missing PARP-1 gene, we employed post-transcriptional gene silencing technology by RNA interference. Specifically, we utilized small interfering RNA (siRNA) to silence PARP-1 gene and hypothesized that the heat shock response is negatively modulated by PARP-1 activation in fibroblasts; therefore siRNA mediated PARP-1 inhibition would lead to augmentation of the heat shock response.

## Material and methods

### Cell culture

Mouse fibroblasts from wild-type mice were created by immortalization by a standard 3T3 protocol [28]. Unless noted otherwise, all reagents were from Sigma-Aldrich (St. Louis MO). Cell monolayers were grown at 37°C in 5% CO_2 _air in Dulbecco's modified Eagle medium (DMEM) (Gibco Technologies, Grand Island, NY) containing 10% fetal bovine serum (FBS), penicillin (100 U/ml), and streptomycin (100 μg/ml). At 75-80% confluence, fibroblasts were subjected to heat shock at 43°C for 45 min followed by recovery at 37°C up to 4 h. If needed, cells were pretreated with PARP inhibitor 1, 5 dihydroxyisoquinoline (DIQ, 100 μM; Sigma, St. Louis, MO) for 45 min in all experiments.

### Nuclear protein extraction

All nuclear protein extraction procedures were performed on ice with ice-cold reagents. Cells were washed twice with phosphate-buffered saline (PBS) and harvested by scraping. Cells were pelleted in 1 ml of PBS at 14,000 rpm for 1 min. The pellet was washed twice with PBS and resuspended in lysis buffer [10 mM Tris-HCl (pH 7.8), 10 mM KCl, 1 mM ethylene glycol tetra acetic acid (EGTA), 5 mM MgCl_2_, 1 mM dithiothreitol (DTT), and 0.5 mM phenylmethylsulfonyl fluoride (PMSF)]. The suspension was incubated on ice for 15 min and Nonidet P-40 was added followed by centrifugation at 4°C at 2,000 rpm for 5 min. The supernatant was discarded and the cell pellet was dissolved in extraction buffer (20 mM Tris-HCl, pH 7.8, 32 mM KCl, 0.2 mM EGTA, 5 mM MgCl_2_, 1 mM DTT, 0.5 mM PMSF and 25% v/v glycerol) was added to the nuclear pellet and incubated on ice for 15 min. Nuclear proteins were isolated by centrifugation at 14,000 rpm at 4°C for 10 min. Protein concentrations of the resultant supernatants were determined using the Bradford assay. Nuclear proteins were stored at -70°C until used for electromobility gel shift assays (EMSA).

### EMSA

EMSA were performed as previously described [29]. An oligonucleotide probe corresponding to an HSF-1 consensus sequence (5'-GCC TCG ATT GTT CGC GAA GTT TCG-3') was labeled with γ-[^32^P] ATP using T4 polynucleotide kinase (Promega) and purified in Bio-Spin chromatography columns (GE Healthcare, Buckinghamshire, UK). For each sample 4 μg of nuclear proteins were incubated with Bandshift buffer (10 mM Tris, 40 mM KCl, 1 mM (ethylene diamine tetra acetic acid) EDTA, 1 mM DTT, 50 ng/ml poly d(I-C), 10% glycerol) at room temperature with subsequent addition of the radiolabeled oligonucleotide probe for 30 min. Protein-nucleic acid complexes were resolved using a nondenaturing polyacrylamide gel consisting of 5% acrylamide (29:1 ratio of acrylamide: bisacrylamide) and run in 0.25 X Tris/Borate/EDTA (TBE) (45 mM Tris, 45 mM boric acid, 1 mM EDTA) for 1 h at 30 mA constant current. Gels were transferred to Whatman 3 MM paper, dried under a vacuum at 80°C for 1 h, and used to expose to X-ray film at -70°C with an intensifying screen.

### Real-time reverse transcriptase-PCR analysis

Fibroblasts were subjected to heat shock at 43°C for 45 min followed by recovery at 37°C for 120 min. Cells were harvested in 1 ml of TRI-Reagent as directed by the manufacturer (Molecular Research Center, Cincinnati, OH). Bromochloropropane was used for the extraction. The final RNA pellet was dissolved in nuclease - free water and quantified using a GeneQuant Pro UV spectrophotometer (GE Healthcare). Extracted RNA (1 μg/reaction) was converted to single-stranded cDNA in a 20 μl reaction using the Reverse Transcriptase System Kit (Promega) as directed by the manufacturer. The mixture was heated to 70°C for 10 min, maintained at 42°C for 30 min, and then heated to 95°C for 5 min using a Gene Amp PCR System 9700 (Applied Biosystems, Foster City, CA). TaqMan Gene Expression Assays for HSP-70 (GENBANK accession no. NM 010479), 18 S RNA (endogenous control) and real-time PCR reagents were purchased from Applied Biosystems (Foster City, CA). Reaction mixtures for PCR were assembled as follows: 10 μl TaqMan Universal PCR Master Mix, 1 μl of each Gene Expression Assay mix, 1 μl cDNA template and 7 μl of water. PCR reactions were performed in an Applied Biosystems thermocycler 7300 Real Time PCR System by incubating at 50°C for 2 min, 95°C for 10 min, 95°C for 15 s, and 60°C for 1 min; the two final conditions were repeated for 40 cycles. Each sample was assayed in duplicate and the values were averaged. A ΔΔ C_t _relative quantification method was used to calculate mRNA levels for HSP-70 in the samples. Results were normalized relative to 18 S rRNA expression.

### SiRNA-mediated inhibition of PARP expression

Stealth small interference RNA (siRNA) sequences for PARP (sequences) were designed using Invitrogen on line software (Block-iT™RNAi Express) to target PARP-1 mRNA (accession number NM007415). Small interfering RNA (siRNA)-mediated silencing of the PARP-1 gene was performed using 21-bp siRNA duplexes purchased from Ambion (Austin, TX). The coding strand for PARP-1 siRNA was 5'-AUG UCG GCA AAG UAG AUC CCU UUC C-3'. An unrelated siRNA sequence (catalog number 12935-113) was used as a control. In this experiment, cells were incubated for 6 h and transfected at approximately 40% confluency with 20nm siRNA duplexes using Lipofectamine™2000 (Invitrogen, Carlsbad, CA) according to the manufacturer's instructions. All the experiments were performed 18 h after transfection. The efficiency and specificity of siRNA gene knockdown of PARP-1 was determined by real time PCR for PARP-1 mRNA and Western blotting for PARP-1 expression.

### Western blot analysis

Western blot analyses were performed as previously described [29]. Briefly, whole cell lysates containing 30 μg of protein were boiled in equal volumes of loading buffer (125 mM Tris, pH 6.8, 4% sodium dodecyl sulfate (SDS), 20% glycerol, and 10% β-mercaptoethanol). Proteins were separated on 8-16% polyacrylamide gels and subsequently transferred to polyvinylidene difluoride (PVDF) membranes (GE Healthcare, Buckinghamshire, UK). For immunoblotting, membranes were blocked with 5% non-fat dried milk in PBS for 1 h. Primary antibodies against the inducible isoform of HSP-70 (Stressgen, Victoria, BC, Canada) were applied at 1:2500 dilution for 1 h. After washing twice with PBS containing 0.5% Tween 20 (PBST), secondary antibody (horse radish peroxidase-conjugated goat anti- rabbit immunoglobulin G, Stressgen, Victoria, British Columbia) was applied at 1:4,000 dilution for 1 h. Blots were washed in PBST thrice for 10 min, incubated in Enhanced Chemiluminescence Reagent (GE Healthcare), and used to expose X-ray film (GE Healthcare).

### Immunoprecipitation

Nuclear extracts were incubated with normal mouse IgG-AC (20 μl Santa Cruz, sc-2343) and incubated for 30 min at 4°C. Anti-PARP antibody (10 μl Biomol, SA-250) or HSF-1 antibody (Stressgen, SPA-950) and non-specific IgG was added to the supernatant for 1 h at 4°C. Thereafter, protein A/G PLUS-Agarose beads were added (20 μl Santa Cruz Biotechnology, sc2003) and the samples were incubated overnight at 4°C. Beads were washed three times in volume 1xPBS and resuspended in 2 X SDS- polyacrylamide gel electrophoresis (PAGE) sample buffers and analyzed by 8% SDS-PAGE. The proteins were then transferred onto PVDF membranes (GE Healthcare, Buckinghamshire, UK). The membranes were blocked in 1X PBST containing 5% nonfat dry milk and incubated with an HSF-1 antibody (Stressgen, SPA-950) or Anti-PARP antibody (10 μl Biomol, SA-250). The membranes were washed and incubated with a polyclonal rabbit anti -rat antibody conjugated to horseradish peroxidase (Stressgen, SAB-200). Immunoreaction was visualized by chemiluminescence.

### Data analysis

All values in the figures and text are expressed as mean ± SEM. The results were examined by analysis of variance followed by the Bonferroni's correction post hoc *t *test. A *p*-value less than 0.05 were considered significant.

## Results

### Inhibition of PARP-1 expression by RNA interference augments HSP-70 protein expression

**Figure 1:**
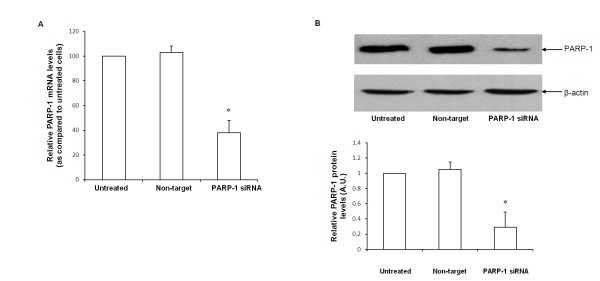
**Naïve, non target siRNA and PARP-1 siRNA transfected cells were tested for PARP-1 gene expression 18 h after transfection**. Figure 1A-Quantitative Real time PCR of PARP-1 mRNA normalized for 18 S mRNA expression. Figure 1B-Representative Western blot analysis for PARP-1 expression in naïve, non target siRNA and PARP-1 siRNA transfected cells (* Represents *p *< 0.05 *versus *naïve cells at the same time point).

To investigate the biological consequences of PARP-1 activation and its effect on the heat shock response, we employed a siRNA based approach to selectively inhibit PARP-1 expression. As a first step, we treated fibroblasts with various siRNA concentrations (10 nm to 100 nm) and evaluated PARP-1 mRNA and protein expression 18 h after transfection. The lowest concentration of PARP-1 siRNA resulting in efficient PARP-1 gene knockdown, as evidenced by a decrease in PARP-1 mRNA and protein expression, was 20nm (Figure 1A and 1B). This concentration was employed in all subsequent experiments.

**Figure 2:**
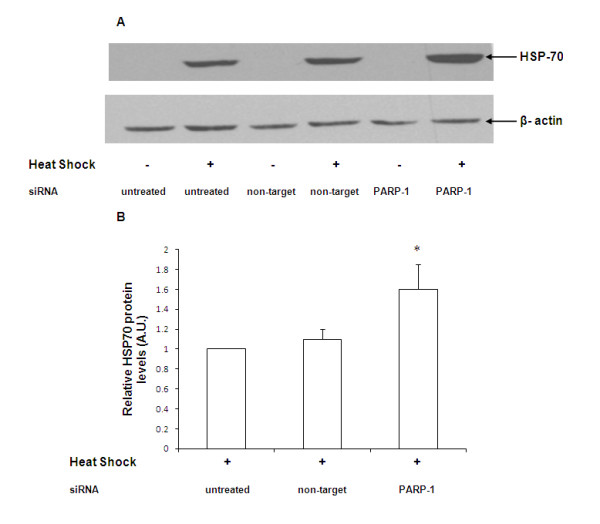
**Representative Western blot analysis for HSP-70 expression in naïve, non target siRNA and PARP-1 siRNA transfected cells**. Radiographs of Western blot analyses in whole cell extracts are representative of three similar separate experiments. Cells were subjected to heat shock at 43°C for 45 min followed by recovery at 37°C for 4 h. In panel 2B the Western blot was quantitated by PhosphorImager analysis and the mean ± SEM plotted from three independent experiments (* Represents *p *< 0.05 *versus *heat shocked naïve cells at the same time point).

Cells were transfected with siRNA for 12 h, subjected to heat shock for 45 min and allowed to recover for 4 h. The expression of HSP-70 was determined by immunoblotting. After heat shock, naïve cells demonstrated a significant increase in HSP-70 protein expression (Figure 2A). HSP-70 protein expression in cells transfected with non-target siRNA was comparable to naïve cells after heat shock (Figure 2A and 2B). Using siRNA to silence PARP-1, we observed that HSP-70 protein expression in PARP-1 siRNA-transfected cells was markedly upregulated as compared to naïve or non-target siRNA transfected cells (Figure 2A and 2B). These data support the view that PARP-1 gene silencing leads to augmentation of HSP-70 protein expression after heat shock.

### HSP-70 mRNA expression is increased with inhibition of PARP-1 expression

**Figure 3:**
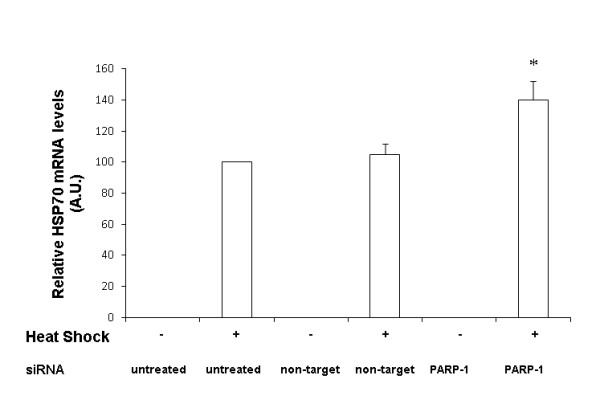
**Quantitative Real time PCR of HSP-70 mRNA normalized for 18 S mRNA expression**. Naïve, non target siRNA and PARP-1 siRNA transfected cells were subjected to heat shock at 43°C for 45 min followed by recovery at 37°C for 1 h (*Represents *p *< 0.05 *versus *heat shocked naïve cells at the same time point).

To further ascertain that PARP-1 inhibition augments the heat shock response by enhancing HSP-70 gene transcription, we next determined its effect on HSP-70 mRNA using real time RT-PCR. HSP-70 mRNA was examined at 60 min after heat shock in transfected and naïve wild-type cells. After heat shock, both naïve cells and wild-type cells transfected with non-target siRNA had comparable HSP-70 mRNA levels (Figure 3). In contrast, PARP-1 directed siRNA led to a significant increase in HSP-70 transcripts as compared to cells transfected with non-target siRNA levels (140 ± 12 vs. 105 ± 7 A.U.). These data reinforce the notion that PARP-1 knockdown leads to a robust heat shock response as evidenced by increase in HSP-70 mRNA and protein expression in PARP-1 knockdown cells (Figure 3).

### HSF-1 DNA-binding activity is increased with inhibition of PARP-1

**Figure 4:**
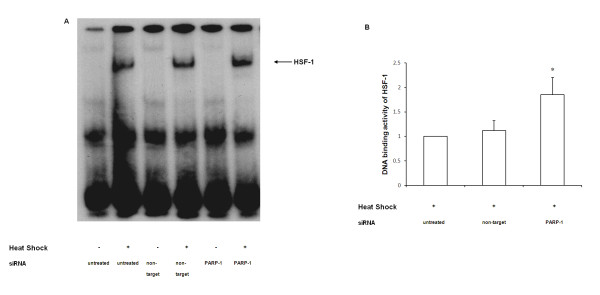
**A. DNA binding of HSF-1 after heat shock in naïve, non target siRNA and PARP-1 siRNA transfected cells**. Cells were subjected to heat shock at 43°C for 30 min followed by recovery at 37°C for 45 min. Autoradiograph of EMSA for HSF-1 is representative of 3 similar separate experiments. **B**. Mean ± SEM of scanned densitometry data demonstrating the effect of PARP-1 silencing on DNA binding of HSF-1 obtained from three independent experiments.

HSF-1 is a key transcription factor that regulates HSP-70 gene expression [22,23]. Hence, we sought to determine if PARP-1 knockdown increased DNA binding of HSF-1. We subjected both naïve and siRNA transfected cells to heat shock and evaluated DNA binding of HSF-1 by EMSA. After heat shock, both naïve and non-target siRNA transfected cells demonstrated comparable DNA binding activity of HSF-1. Using EMSA, we found that nuclear extracts from cells transfected with PARP-1siRNA displayed increased binding of HSF-1 to nuclear DNA as compared with naïve and non-target transfected cells (Figure 4A and 4B). Collectively, these experiments suggest that PARP-1 negatively modulates the heat shock response i.e. knockdown of PARP-1 led to augmentation of the heat shock response by increasing HSF-1 activation, subsequently leading to increased HSP-70 gene expression.

### PARP-1 interacts with HSF-1

Because previous studies reported that PARP-1 may regulate transcription factors by a direct physical association [7,8,30] we next explored the possibility of a protein-protein interaction between PARP-1 and HSF-1.

**Figure 5:**
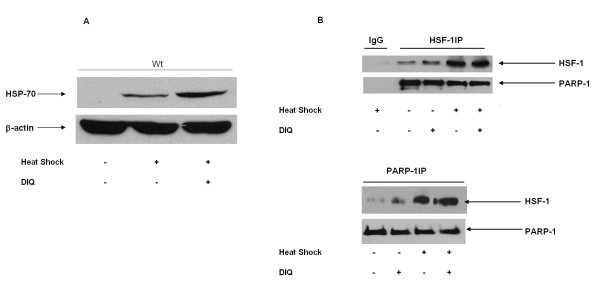
**A. Representative Western blot assay for HSP-70 expression in wild-type (wt) cells**. Radiographs of Western blot analyses in whole cell extracts are representative of three similar separate experiments. Cells were subjected to heat shock at 43°C for 45 min followed by recovery at 37°C for 4 h in the presence or absence of DIQ treatment (100 μM). **B**. Cells were subjected to heat shock at 43°C for 45 min followed by recovery at 37°C for 45 min in the presence or absence of 1, 5-DIQ treatment (100 μM). Representative radiograph from co-immunoprecipitation experiments showing PARP-1/HSF-1 interaction. Nuclear lysates were immunoprecipitated with either HSF-1or PARP-1 antibody and subsequently probed with anti-HSF-1or PARP-1 antibody.

First, we confirmed that the modulation of the heat shock response by DIQ, a PARP-1 inhibitor is similar to the increase noted after siRNA mediated PARP-1 inhibition. Wild-type cells were subjected to heat shock at 43°C for 45 min and allowed to recover for 4 h. The expression of HSP-70 was determined by immunoblotting. DIQ pretreatment of wild-type cells not subjected to heat shock did not induce HSP-70 protein expression (data not shown). After heat shock, DIQ pretreated wild-type cells demonstrated significant increase in HSP-70 expression as compared to untreated cells (Figure 5A). Thus, similar to siRNA mediated PARP-1 inhibition, pharmacologic inhibition of PARP-1 also increases HSP-70 protein expression.

To determine if there is protein-protein interaction between PARP-1 and HSF-1, nuclear lysates were immunoprecipitated with antibodies against HSF-1. As shown in Figure 5B, HSF-1 was efficiently immunoprecipitated with HSF-1 antibody and no signal was observed when mouse IgG was used as a control for immunoprecipitation. Immunoblotting of the HSF-1immunoprecipitated proteins with PARP-1 antibody demonstrated the presence of PARP-1 suggesting that HSF-1and PARP-1 physically interact with each other. Wild-type cells treated with DIQ (PARP-1inhibitor) that had not been subjected to heat shock demonstrated a slight increase in nuclear HSF-1 content as compared to control cells. Cells pretreated with DIQ and subsequently exposed to heat shock demonstrated increased HSF-1 binding to PARP-1 as compared to untreated control heat shocked wild-type cells (Figure 5B).

To confirm the results obtained by HSF-1 immunoprecipitation, we conducted the reverse experiment by immunoprecipitating with a PARP-1 antibody and subsequent analysis of the immunoprecipitate for HSF-1. Similar to our results above, cells pretreated with a PARP-1inhibitor demonstrated a slight increase in HSF-1 content as compared to control cells. Analogous to our finding above, nuclear lysates exposed to a PARP-1 inhibitor prior to heat shock demonstrated increased HSF-1 binding to PARP-1 in comparison to cells that were not exposed.

Thus, in this study we demonstrate that PARP-1 physically interacts with HSF-1 in resting and heat shocked cells. Furthermore, these immunoprecipitation studies confirm that exposure to heat shock increases the nuclear HSF-1 binding to PARP-1.

## Discussion

PARP-1 catalyses the covalent attachment of ADP-ribose units on to the γ carboxyl group of glutamate residues in acceptor proteins including PARP-1 itself [5,31,32]. Each of these ADP-ribose units has an adenine moiety capable of base stacking and hydrogen bonding, along with two phosphate groups that carry negative charges. These polymers cause profound changes in the structure and function of key proteins that respond to DNA damage. For example, proteins like histones, topoisomerases I and II, and DNA helicases undergo poly (ADP-ribosylation) and help in regulation of chromatin structure and genomic integrity [3-5]. However, a growing body of evidence suggests that in addition to its regulation of chromatin structure, PARP-1 also plays a key role in gene-specific transcription.

PARP-1 regulates gene-specific transcription by two possible mechanisms. Kim *et al*. demonstrated that PARP-1 binds to specific nucleosomes and leads to formation of compact, transcriptionally repressed chromatin structures [33]. Upon activation, PARP-1 is poly (ADP-ribosylated) and dissociates from chromatin leading to formation of decondensed, transcriptionally active euchromatin structures. It has also been proposed that PARP-1 is part of a nucleosome complex along with a histone variant macroH2A (mH2A). At baseline conditions, mH2A and an inactive PARP-1 are associated with the HSP-70 promoter. Upon heat shock, HSP-70 promoter bound PARP-1 is released to activate HSP-70 transcription [34]. Hence, siRNA mediated knockdown of either PARP-1 or mH2A1 downregulated transcription of HSP-70 gene. This observation was complemented by another study that demonstrated chromosomal "puffing" with increased HSP-70 gene expression in *Drosophila *salivary glands after heat shock [35]. It was proposed that PARP dissociates chromatin proteins at induced chromosomal loci, thus allowing increased transcription of target heat shock genes. In contrast to our study, treatment with an inhibitor of PARP activity reduced puffing and consequently decreased transcription of heat shock genes following heat shock. Our data are consistent with our previous *in vivo *study of myocardial ischemia-reperfusion injury, where we demonstrated that mice with genetic ablation of PARP-1 exhibit significant cardioprotection, associated with enhanced upregulation of HSF-1 DNA binding in the heart [26]. The reasons for the different response of dipterans and mammals to PARP-1 inhibition are unknown. It is plausible that factors including signaling mediators have evolved different effector proteins that can affect the heat shock response and its regulation by PARP-1.

Another potential mechanism which may be more relevant to this study entails the role of PARP as a gene specific transcription enhancer/promoter binding cofactor activity that can enhance or inhibit gene expression. PARP-1 has been shown to interact with the transcription factors NF-κB, HTLV Tax-1 and RAR and this was associated with increased expression from dependent promoters [7-9]. Similarly, it has been suggested that PARP-1 is a co-transcription factor for the mammalian *achaete-scute *homologue (MASH) gene. PARP-1 is present in an inactive state as part of a co-repressor complex. Upon activation, PARP-1 is required for the dismissal of the co-repressor complex, and a second subsequent event leads to activation of the target MASH gene [36].

Our experiments indicate that PARP-1 modulates the heat shock response by functioning as a repressing factor of HSP-70 gene expression. We provide two lines of evidence in this regard. First, in this study we demonstrated that knockdown of PARP-1 gene increased HSP-70 gene expression as evidenced by increased DNA-binding activity of HSF-1, HSP-70 mRNA and protein expression. Secondly, using co-immunoprecipitation we demonstrated that PARP-1 may also regulate HSF-1 activation through direct interaction with this transcription factor. Protein-protein interaction is recognized as a mechanism for PARP-1 to function as a specific transcriptional co-activator of NF-κB [37].

Fossati *et al*. similarly documented increased HSP-70 expression in murine PARP-1 deficient fibroblasts as compared to wild type fibroblasts [27]. In contrast to our findings, this study was unable to detect PARP-1 and HSF-1 interaction by co-immunoprecipitation studies. While the cell type utilized in the two studies was remarkably similar, the duration of heat shock was different. In our study, the cells were subjected to 45 min of heat shock in comparison to 30 min in the study by Fossati *et al. *[27]. Other differences that could lead to different results may be the antibody type and protocol design for immunoprecipitation studies.

Other studies have also proven that PARP-1 modulates transcription by direct interaction with AP2 [38], Oct-1[39], YY-1 [40] and TEF-1 [41]. PARP has been also shown to alter RNA polymerase II dependent transcription [42] and to effectively prevent and reverse p53 binding to the palindromic p53 consensus sequence [43]. Before HSF-1 is activated there are a series of processes that involve phosphorylation, translocation from the cytosol to the nucleus, formation of a trimer, binding to heat shock elements (HSE), and initiating HSP-70 gene expression [44-46]. It has been postulated that addition of long ADP-ribose tails to transcription factors can disable or dissociate the binding of transcription factors to their DNA recognition sites, also in part by electrostatic repulsion. This modification results in inhibition of transcription. Poly (ADP-ribosylation) of transcription factors prevents their binding to DNA. On the contrary, inhibition of PARP-1 enables the binding of the transcription factors to their specific DNA sites [5]. Thus, it is possible that both physical interaction with PARP-1 and poly (ADP-ribosylation) of HSF-1 reduce the availability of HSF-1 to initiate transcription. The increase in HSF-1 content, albeit inactive with DIQ pretreatment further reinforces the notion that PARP-1 represses HSP-70 gene transcription. Further studies need to be conducted to understand the precise mechanism as to how and where PARP-1 regulates HSF-1 activation.

In conclusion, our results indicate that PARP-1 serves as a repressing factor of the heat shock response by regulating the expression of HSP-70. Both protein-protein interaction and catalytic activity of the PARP protein play a key role in modulation of the heat shock response.

## Competing interests

The authors declare that they have no competing interests.

## Authors' contributions

HS carried out the molecular studies. JG carried out the RT-PCR assays. BZ, RA conceived the study. RA, RLD participated in the design of the study. All authors read and approved the final manuscript.
